# A scoping review of TSR analysis in colorectal cancer: implications for automated solutions

**DOI:** 10.3389/or.2025.1605383

**Published:** 2025-10-28

**Authors:** Felix Anne Dikland, Cyrine Fekih, Marius René Jacques Wellenstein, Ricella Souza da Silva, Raquel Machado-Neves, João Fraga, Domingos Oliveira, Diana Montezuma, Isabel Macedo Pinto, Jonathan Woodburn

**Affiliations:** ^1^ Department of Research and Development, WSK Medical, Amsterdam, Netherlands; ^2^ WSK Medical, Amsterdam, Netherlands; ^3^ IPATIMUP Diagnostics, IPATIMUP-Institute of Molecular Pathology and Immunology of Porto University, Porto, Portugal; ^4^ Department of Pathologic Anatomy, Hospital de Matosinhos, Unidade Local de Saúde de Matosinhos, Matosinhos, Portugal; ^5^ Research and Development Unit, IMP Diagnostics, Porto, Portugal; ^6^ Cancer Biology & Epigenetics Group, Research Center of IPO Porto (CI-IPOP), Portuguese Oncology Institute of Porto (IPO Porto), Porto Comprehensive Cancer Center Raquel Seruca (Porto.CCC), Porto, Portugal; ^7^ IMP Diagnostics, Porto, Portugal

**Keywords:** tumour-stroma ratio, colorectal carcinoma, scoping review, computational pathology, observer variability, prognostic value, protocol standardisation, artificial intelligence

## Abstract

The tumour-stroma ratio (TSR), which refers to the composition of stromal tissue and tumour epithelium of a malignant lesion, is gaining recognition as a promising biomarker in pathology. In 2018, recommendations for quantifying TSR in colorectal carcinoma were published, yet diverse quantification methods are still in use today. To assess the prognostic value of TSR, evaluate the impact of scoring variations, and explore efforts to automate TSR quantification, a scoping review was conducted. A total of 950 articles were identified through PubMed and Scopus, of which 76 met the inclusion criteria for this review. Of these, 56 employed manual scoring methods, while 20 utilised semi-automated or fully automated TSR quantification techniques. The TSR has been consistently identified as a strong prognostic indicator for disease-free survival. Its association with poor prognosis may be linked to its correlation with metastatic status, perineural invasion, and vascular invasion in stroma-high lesions. Variability in TSR scoring protocols was most evident in the selection of the region of interest and the type of histological specimen, both of which had a direct impact on final TSR scores. Moreover, significant inter-observer variability was observed in manual semi-quantitative TSR assessments, with Kappa scores ranging from 0.42 to 0.88. Automated TSR scoring pipelines have been proposed to standardise scoring protocols and reduce inter-observer variability. Deep learning models have demonstrated promising results, with pixel-wise and patch-wise accuracies exceeding 95%. Even though deep learning approaches have shown high performance, discrepancies remain, as evidenced by Kappa scores ranging from 0.239 to 0.472. In conclusion, the variation in TSR scoring protocols, along with a wide range of inter-observer variability, limits the broader clinical application of TSR. While automated TSR quantification methods show promise, they are still in the early stages, particularly in relation to region of interest selection and stratifying patients into risk categories. As these methods evolve, adjustments to TSR scoring cut-off values may be necessary to improve consistency. This scoping review highlights the prognostic significance of TSR in colorectal carcinoma while emphasizing the challenges posed by variability in scoring methods and the need for further advancements in automated quantification.

## 1 Introduction

Staging of cancer is crucial for predicting a patients prognosis and developing a treatment plan. In colorectal cancer (CRC) staging is performed according to the American Joint Committee on Cancer (AJCC) TNM system (1). Besides the TNM staging, there are additional CRC histological features that hold prognostic relevance for CRC patients (2–4). Histological characteristics that are currently considered to be reported in routine diagnostics as core elements are: histologic type and grade; presence of perforation; distance to surgical margins; lymphovascular and perineural invasion; tumour budding; tumour deposits, and treatment response. There are other histology factors associated with prognosis that are not yet included in the recommendations for routine diagnostics, such as the tumour growth pattern and immune response (5).

An important biomarker that has gained increasing attention in recent years is the tumour-stroma ratio (TSR), which refers to the composition of stromal tissue and tumour epithelium of a malignant lesion (6). Studies have suggested that a high stromal content is associated with a worse patient prognosis, as the stroma can promote tumour progression and possibly increase resistance to treatment (7–10). The prognostic value of TSR has been demonstrated not only for CRC, but also for other cancer types, namely, breast, oesophageal and lung (11–15).

Despite the wide support of the prognostic power of TSR, it has not yet been implemented in routine diagnostics. However, it has been reported that the TNM Evaluation Committee and the CAP have acknowledged its potential for integration in the TNM staging system (16,17). Moreover, a large prospective multicentre European study has recently validated TSR as an independent prognosticator for disease-free survival (DFS) in stage II-III colon cancer (CC) patients (18). As such, TSR is an emerging and promising histological biomarker with the potential to serve as a reliable prognostic indicator in CRC.

Notwithstanding the emerging prognostic significance of TSR in CRC, several challenges can be identified. The absence of a universally accepted methodology for TSR assessment is the greatest hurdle. In 2018 a study by van Pelt et al. on the procedure and recommendations of TSR scoring was released, which the majority of recent studies have adhered to (9). Nonetheless, there is still a variety in scoring methods concerning the region of interest (ROI) and histological specimen type (19–21). This variability complicates comparisons across studies and limits the reproducibility of findings. Compounding this issue is the challenge of intra-tumoural heterogeneity, which causes TSR scores to differ across ROIs within a single slide. Protocols that use the highest stroma-containing field as the decisive TSR score, mitigate this effect by focusing on the region with maximal stromal content. Anyhow, some degree of variability may still arise, for instance if the most stroma-rich region is not represented on the available slides. This is particularly true when assessing TSR in biopsies. Also, the semi-quantitative nature of TSR scoring makes it inherently subjective.

Even though the score is relatively simple to perform, it requires the careful identification of the ROI and accurate evaluation of the TSR within the constraints set by Van Pelt et al. (9). The impact of these challenges is particularly evident when developing computational models to perform this task, as variations in slide selection, ROI identification, and subjective interpretations introduce significant variability in the data. This variability can complicate the training and validation of algorithms, potentially limiting their accuracy and generalisability across diverse clinical settings. On the other hand, machine learning (ML) tools themselves can also be the solution, as these tools have the potential to standardise TSR scoring by automating the process and defining ROIs consistently, minimising subjective biases and ultimately enhancing reproducibility and efficiency.

Intended and unintended deviations from the protocol inevitably lead to a lowered reliability of the TSR score and might hamper the adoption as a diagnostic tool in clinical practice. Despite promising evidence of high inter-observer variability prior to publishing of the protocol by Van Pelt et al., there has not been an overview of observer variability scores since the introduction of said protocol. Moreover, it is uncertain to what extend deviations from the TSR scoring protocol influence the TSR score. The effects of protocol changes to the final TSR score must be mapped in detail to identify shortcomings of existing automated scoring pipelines. Mediators that cause stroma-high lesions to have a poor prognosis need further investigation. To explore these concerns and provide a robust understanding of the protocol for development of new automated quantification methods, a scoping review was conducted.

The research questions of TSR in CRC were articulated as follows: 1) “What is the prognostic value of TSR, and what are its possible mediators?”; 2) “How do TSR scoring protocols differ, and how do these scoring variations influence the final TSR score?”; and 3) “How reliable is manual TSR scoring, and how well-developed are current automated solutions?”.

## 2 Methods

The study adhered to the Preferred Reporting Items for Systematic Reviews and Meta-Analyses guideline for scoping reviews (PRISMA-ScR) (22). A systematic literature search was performed using the Scopus and PubMed medical databases. Standardisation of TSR scoring in CRC was proposed by Van Pelt et al. in 2018; therefore only studies published from 2018 onwards were included in this review. Relevant papers were identified using the following query, performed on 21st of February 2025: “ (“TSR” OR “tumo*r stroma”) AND (“Colorectal” OR “CRC” OR “colon*” OR “rectal”)”. Studies were excluded if they met any of the following criteria. The article: 1) was not written in English; 2) was a conference paper, abstract-only publication, case study, letter to editor, comment, study protocol or preprint, 3) did not report a stroma content score, or did not correlate it to staging or prognosis, 4) was conducted on animal models or in-vitro, 5) did not include CRC-diagnosed subjects. Exclusion was performed by two independent observers. Disagreement was resolved by team discussion.

Included reviews were thoroughly investigated for general concepts and knowledge gaps. Methodological data extraction was performed on original works only, structured around four main topics: 1) the prognostic significance of TSR and its possible mediators causing worse prognosis, 2) variability in TSR scoring protocols, 3) inter- and intra-observer variability in TSR assessment, and 4) automation of TSR scoring.

These topics led to the creation of a data-charting form developed by two reviewers that was updated iteratively. Data was extracted by a single investigator and reviewed by a second. For investigation of prognostic value, conclusions of studies correlating TSR to survival outcomes, TNM staging, and local infiltration were charted. Variability in the scoring protocol was investigated by describing individual factors of the scoring method that influence the final TSR. This included ROI location, ROI size, lens magnification, histological specimen type, mode of automation and a mathematical representation of the scoring protocol. For observer variability assessment, the agreement metric is reported as well as the TSR evaluation task, which can either be ROI selection and TSR estimation combined or TSR estimation alone in a given ROI. Charted information on automated TSR evaluation include the algorithm type, its mode of automation, the ROI used for scoring, its tissue identification performance metrics and its agreement with a manual scoring process. Quantitative data is structured in tables and qualitative data is presented in a narrative format.

## 3 Results

**FIGURE 1:**
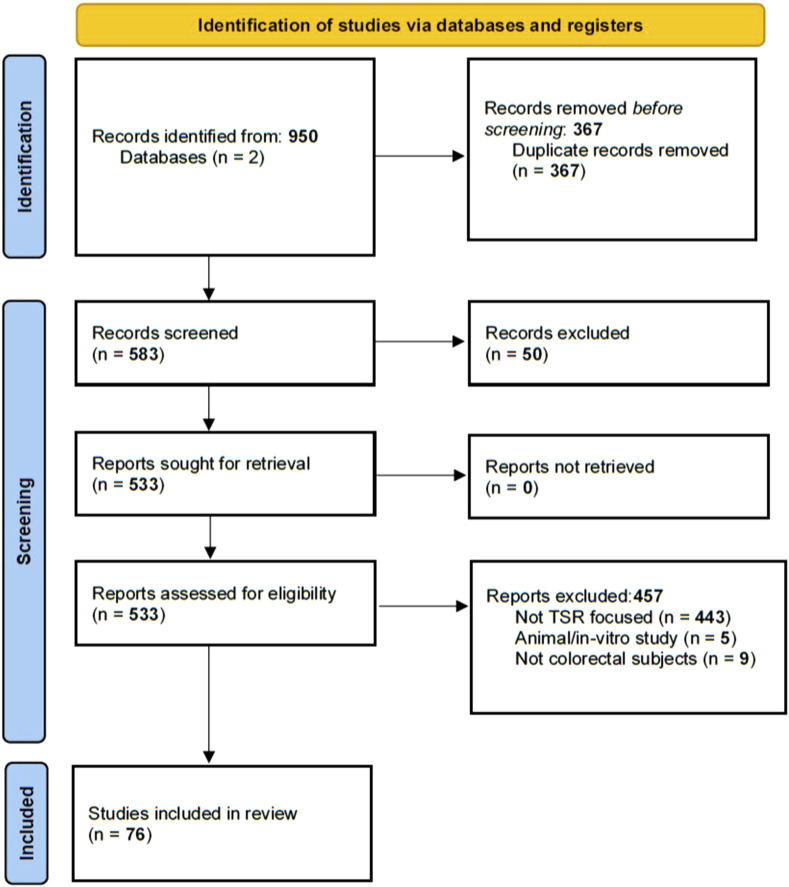
A flowchart showing the exclusion from the Pubmed and Scopus database.

A total of 411 papers were identified through the PubMed search. The Scopus search identified 539 articles, of which 172 were unique. This resulted in a total of 583 studies for initial screening. Based on title and abstract review, 50 papers were excluded. Following full-text assessment, an additional 457 articles were excluded, leading to the final inclusion of 76 papers, as seen in Figure 1 and Supplementary Table S1. Of the 76 included articles 56 adopted a manual approach to evaluate the TSR score. Of the remaining 20 articles, 5 adopted fully automated solutions, 14 used semi-automated solutions and a single paper did both. The percentage of stroma-high subjects included in a study range from 2% to 86% with a median of 37%.

### 3.1 Prognostic value of TSR

#### 3.1.1 As an individual biomarker

The correlation between TSR and DFS and OS was generally found to be significant. Twenty-nine studies have analysed DFS as primary or secondary outcome and 25 of these, including three meta-analyses, demonstrated a significantly worse prognosis for patients with high stroma content, as seen in Table 1. Five studies did not report a significant association with TSR (21,23–26). The association between TSR and OS followed an equivalent trend. Among the 35 studies investigating OS, 26 studies, including the aforementioned meta-analyses, demonstrated a significantly higher mortality risk in patients with high stroma content. Additionally, ten studies, including the recent prospective multicentre UNITED study, did not reach statistical significance but still reported a trend towards worse prognosis in high-stroma tumours (18). It should be noted that the UNITED study was specifically powered for 3-year DFS with a 5-year OS as a secondary outcome. Three articles did not find a trend of worse prognosis with high stroma. These studies had a dataset with a very low amount of stroma-high subjects, or investigated only stage I or IV subjects (26–28).

**TABLE 1:** Studies investigating the association of stroma high lesions with OS and DFS. Significant correlations are denoted with “yes”, and non-significant correlations are denoted with “no”.

Author	Year	Number of subjects	Stroma-high subjects [%]	Overall survival	Disease free survival	Cancer specific survival
Kristensen MP (65)	2025	497	31%	yes	yes	
Carvalho R (56)	2025	1,317	25%	yes	yes	yes
Zhao Y (24)	2025	179	20%	no	no	
Hong SA (48)	2024	323	33%	yes		
Sinicrope FA (52)	2024	380			yes	
Fekete Z (27)	2024	74	7%	no		
Jakab A (29)	2024	185	35%	yes		
Polack M (18)	2024	1,388	31%	no	yes	
Inoue H (54)	2023	200	50%	yes	yes	
Magnusson MI (80)	2023	2,162	26%	yes		
Pyo JS^a^ (15)	2023	2,999	35%	yes	yes	
Strous MTA (73)	2023	201	29%		yes	
Aboelnasr LS (31)	2023	103	65%	yes	yes	
Yang J (47)	2022	1,010	29%	yes		
Wang Q (81)	2022	114	41%	yes	yes	
Strous MTA (10)	2022	578	27%		yes	
Jin HJ (46)	2022	487	20%		yes	yes
		143	2%	no	no	
Zhao Z (26)	2021	179	24%	yes	yes	
		174	39%	no	no	
Fan S (25)	2022	207	45%		no	
Ravensbergen CJ (75)	2021	333	33%	yes		
Jones HJS (68)	2021	143	8%		yes	
Smit MA (66)	2021	246	44%	no	yes	
Gao J^a^ (8)	2021	4,857	25%	yes	yes	
Miller S (33)	2021	253	35%	no		
Li T (69)	2021	996	36%	yes		
Zhu Y^a^ (82)	2021	5,408	32%	yes	yes	yes
Kang G (34)	2021	266	30%	yes	yes	
Zhang Y (23)	2021	147	12%	no	no	
Dang H (28)	2020	223	35%	no		
Zhao K (53)	2020	499	28%	yes		
Zengin M (57)	2020	172	41%	yes	yes	
Martin B (35)	2020	206	35%	yes		
Park JH (59)	2020	115	22%			yes
Van Wyk HC (38)	2019	952	24%	yes		
Zengin M (83)	2019	88	41%	yes	yes	
Zunder M (84)	2019	1,103	30%	yes	yes	
Den Uil SH (21)	2019	107	45%		no	
Sandberg TP (60)	2019	201	48%	no	yes	
Geessink OGF (17)	2019	129	33%	no		no
Eriksen AC (85)	2018	169	29%	yes	yes	
Zunder SM (43)	2018	1,212	28%	yes	yes	
Huijbers A (44)	2018	965	33%		yes	
Hansen TF (37)	2018	65	51%	yes	yes	
Hutchins GGA (49)	2018	1800	75%	yes		

^a^ These studies included a meta-analysis for associating high tumour stroma ratio with OS, and DFS.

The association of TSR with T and N stage was also appraised. Out of 29 studies that mention the T-status stratified by TSR, eleven studies showed a significantly higher T-status for subjects with stroma-high lesions. Of the 23 studies mentioning the N-status, 14 showed a significant correlation of stroma-high lesions with positive lymph node status, as seen in Table 2. Additionally, high stroma seems to be correlated with distant metastases. All eight studies investigating the association of M-status or distant metastasis free survival with TSR found a significantly higher rate of distant metastases in subjects with stroma-high lesions (29–36).

**TABLE 2:** Studies investigating the association of stroma high lesions with increased T-status and positive N-status. Significant correlations are denoted with “yes”, and non-significant correlations are denoted with “no”.

Author	Year	Number of subjects	Stroma-high subjects [%]	T-status	N-status
Kristensen MP (65)	2025	497	31%	yes	
Pujani M (86)	2024	65	45%	yes	no
Unal Kocabey D (87)	2024	126	49%	no	no
Jakab A (29)	2024	185	35%	yes	yes
Strous MTA (73)	2023	201	29%	no	
Khan AA (88)	2023	40	65%	yes	yes
Fan S (25)	2022	207	45%	no	
Tian W (42)	2023	153	41%	yes	yes
Aboelnasr LS (31)	2023	103	65%	no	yes
Strous MTA (73)	2022	187	41%	no	yes
Wang Q (81)	2022	114	41%	yes	yes
Hu S (89)	2022	66	62%	no	no
Da Silva RMS (32)	2022	390	53%	yes	yes
Jin HY (46)	2022	487	20%	no	yes
Ravensbergen CJ (75)	2021	333	39%	no	no
Smit MA (66)	2021	246	44%	no	no
Miller S (33)	2021	253	35%	no	yes
Li T (69)	2021	996	36%	no	yes
Zhu Y^a^ (82)	2021	4,857	35%	no	no
Kang G (34)	2021	266	30%	no	no
Cai C (74)	2021	149	43%	no	yes
Zengin M (57)	2020	172	41%	yes	
Martin B (35)	2020	206	35%	no	no
Fu M (58)	2020	353	38%	yes	yes
Geessink OGF (17)	2019	129	33%	no	no
Eriksen AC (85)	2018	169	29%	no	
Huijbers A (44)	2018	965	33%	yes	yes
Hansen TF (37)	2018	65	51%	yes	yes
Hutchins GGA (49)	2018	1800	75%	no	

^a^ This study included a meta-analysis for associating high tumour stroma ratio with tumour staging.

Multiple studies have also shown an association of high stroma content with perineural invasion and vascular invasion. The correlation with lymphatic invasion and lymphovascular invasion, when considered as a combined parameter, appears more debatable, as seen in Table 3. The tumour budding score, which is also an independent prognostic factor for poor prognosis, is often reported to be associated with stroma high tumours (23,28,31–33,35,37–39).

**TABLE 3:** Studies that investigated the association of stroma content in lesions with invasion of local microstructures. If a higher stroma content is significantly related to a higher invasion rate, this is denoted with “yes”. If this relation is not found it is denoted with “no”.

Author	Year	Number of subjects	Stroma-high subjects [%]	Perineural invasion	Vascular invasion	Lymphatic invasion	Lympho- vascular invasion
Kristensen MP (65)	2025	497	31%	yes	yes	no	
Jakab A (29)	2024	185	35%	yes		yes	
Strous MTA (73)	2023	201	29%				yes
Khan AA (88)	2023	40	65%	yes			yes
Aboelnasr LS (31)	2023	103	65%	yes			yes
Fan S (25)	2022	207	45%	yes			no
Wang Q (81)	2022	114	41%	no	yes		
Jin HY (46)	2022	487	20%	yes	yes		
Da Silva RMS (32)	2022	390	53%	yes			yes
Miller S (33)	2021	253	35%		no		yes
Li T (69)	2021	996	36%		no	no	
Zhu Y (82)	2021	5,408	32%		yes		
Kang G (34)	2021	226	30%	yes	yes	no	
Dang H (28)	2020	223	35%				no
Zengin M (57)	2020	172	41%	yes		yes	
Martin B (35)	2020	206	35%				no
Eriksen AC (85)	2018	573	29%	yes	yes		
Huijbers A (44)	2018	965	33%		yes	no	
Hansen TF (37)	2018	65	51%	yes	yes	no	

Eleven studies investigated therapy resistance. Of these, six focused on neoadjuvant treatment. Four studies investigated the effectiveness of (chemo)radiotherapy and found that tumours that are classified as stroma-high in preoperative biopsies exhibit less tumour regression on the surgical specimen (37,40–42). Li et al. and Yim et al., were unable to replicate these results (19,20). Adjuvant chemotherapy resistance is reported by Strous et al., who found that DFS significantly improved with treatment in stroma-low subjects, but not for stroma-high subjects (10). The UNITED trial suggested chemo-resistance as a possible explanation for significantly worse DFS in stroma-high subjects despite treatment with adjuvant chemotherapy (18). Additional studies have investigated the added benefit of supplementing chemotherapy with Bevacizumab, with mixed results (43,44). Ravensbergen et al. stated that immune checkpoint inhibitor therapy effectiveness cannot be predicted from TSR alone (45).

#### 3.1.2 As a composite score

By visual estimation, machine learning or transcriptomics, TSR can also be combined with tumour immune micro-environment status to create a composite score. Some studies suggested that combining immune scores with TSR provides a superior prognostic value for DFS and OS, compared to using TSR or immune scores alone (46,47). Ravensbergen et al. reported that this combined biomarker could predict the effectiveness of immune checkpoint inhibitor therapy (45). These results led to the creation of the Glasgow micro-environment score by combining the Klintrup-Mäkinen grade with TSR scores, as seen in Supplementary Table S2. This combined score stratifies subjects into three risk categories each associated with progressively worse prognosis (29,48).

### 3.2 Scoring methods and variation

#### 3.2.1 Tumour stroma quantification and TSR cut-off value

A variety of definitions for stromal content are reported in the literature. Numerous studies denominating their stroma evaluation as “TSR”, make use of different definitions and formulas. These methodological variations are summarised in Table 4.

**TABLE 4:** Different definitions of tumour stroma quantification used in the literature.

Definition	Equation	Names and abbreviations
Tumour area per the total area of stroma and tumour^a^	$\frac{\text{Tumour Area}}{\text{Tumour Area}+\text{Stroma Area}}$	Reported Terms: Tumour-stroma ratio (TSR)^b^, Carcinoma percentage (CP) (15,65,86)
		Reported Terms: Tumour stroma percentage (TSP), Proportion of tumour stroma (PTS)
Stroma area per the total area of stroma and tumour^a^	$\frac{\text{Stroma Area}}{\text{Tumour Area}+\text{Stroma Area}}$	“Stroma-high” (>50%) and “stroma-low” (≤50%) are used to directly assess the relation between stroma and tumour tissue (19,48,73)
Tumour area per the field of view	$\frac{\text{Tumour Area}}{\text{Field of view}}$	Reported Terms: Tumour proportion (TP), Proportion of tumour (PoT) (35,49)
Stroma area per the field of view	$\frac{\text{Stroma Area}}{\text{Field of view}}$	Reported Terms: Stroma proportion (SP), Proportion of stroma (PoS), Tumour stroma percentage (TSP) (30,35)
Tumour per Stroma	$\frac{\text{Tumour Area}}{\text{Stroma Area}}$	Reported Terms: Carcinoma stroma percentage (CSP) (51)

^a^ These equations can be expressed either as a decimal value between 0 and 1 or as a percentage.

^b^ Note that studies may use the term TSR, but apply the formula describing the CSP, or TSP.

For quantification of TSR, tissues other than stroma and tumour are usually excluded from the calculation. In contrast, in tumour proportion (TP) and stroma proportion (SP) these tissues are included (35,49). An example of this tissue inclusion discrepancy is found in the evaluation of smooth muscle. In the TSR evaluation, it is excluded, whereas in TP and SP smooth muscle fibres may be present within the field of view (FOV) (9,40). Another two studies considered lumen and mucin as part of the tumour (34,50). Of note is that, despite the recommendations to exclude smooth muscle from TSR scoring in the 2018 guidelines, none of the included studies have used immunohistochemistry to exclude muscle fibres. This means, in practical terms, only visible bundles of muscularis are excluded from eye-scored TSR estimation, ignoring single remaining muscle fibres and cells (9).

Regarding the cut-off value, based on the recommendations proposed by Van Pelt et al., a tumour is classified as stroma-high if the stromal percentage exceeds 50% and as stroma-low if it is 50% or less (9). The cut-off value of 50% was chosen as it provided maximum discriminative power to distinguish prognostic groups (6). Other studies used self-determined cut-off values. One widely used approach is receiver operating characteristic (ROC) curve analysis, often combined with additional metrics such as Youden Index to find the cut-off point with optimal sensitivity and specificity for predicting prognosis (21,30,33,35,51). Another approach used, is to divide TSR into categories, such as quartiles or quintiles, and then choose a cut-off to dichotomise TSR into two prognostic risk groups (46,52). Alternatively, some studies applied other statistical methods, including maximally selected rank statistics to determine an optimal cut-off for predicting OS or using the median TSR value as cut-off (17,53,54). Cut-off values determined using the previously described methods ranged from 40% to 65.5%.

#### 3.2.2 Influence of specimen type and ROI characteristics

On surgical specimens, the TSR should be quantified on the deepest invasion slide (9). On preoperative biopsies it is not possible to choose the deepest invasion slide, and the size and shape of the specimen might make it impossible to have tumour epithelium in all four cardinal directions. In several studies this has led to the choice of using a smaller hotspot or to quantify TSR in the whole slide area (19,20,40,55). Carvalho et al. showed, using automated TSR scoring and mathematical models, that the hotspot size is correlated to the TSR score. Smaller ROIs typically have a higher maximum stroma percentage. This rule of thumb is explained as a smoothing effect enduced by enlarging the ROI (56). Regardless, using TSR in preoperative biopsies for risk stratification has been shown to predict CSS, OS, DFS, lymph node metastasis and distant metastasis (30,57–59). Additionally, stratification of patients based on TSR in preoperative biopsies has been shown to be significantly associated with neoadjuvant treatment response by Liang et al. (40). Two other studies, with smaller sample sizes, did not replicate this result (19,20).

Calculating the TSR over the whole tumour area has been described to consistently underestimating the stroma content in comparison to scoring the TSR in the perceived highest stroma region (55). This TSR difference can be attributed to the stroma heterogeneity of the lesion. In surgical specimen three ROIs are commonly used 1) whole tumour area, 2) infiltrative edge, and 3) highest stroma region. On average, the TSR measured across the whole tumor is lower than the TSR in the infiltrative edge, which is, in turn, lower than the maximum TSR observed in CRC surgical specimen slides (33,35,51). TSR in any of the three regions of interest is a predictor of poor prognosis, but the optimal cut-off value for risk stratification differs, with $C_{\mathrm{WholeTumour}}<C_{\mathrm{InfiltrativeEdge}}<C_{\mathrm{MaximumStroma}}$ (49,51).

Further to scoring TSR in the primary neoplasm specimen, Ubink et al. investigated the use of TSR in peritoneal metastasis and found a significant correlation between TSR in metastatic site and TSR in the primary tumour (36). Also, combining the TSR value in the lymph node with the TSR determined in the primary tumour yielded higher prognostic value than TSR of the primary tumour alone. This was found when reclassifying a subject as stroma-high if TSR in the affected lymph node was higher than 50% regardless of TSR in the primary tumour (10). Although a significant correlation between TSR in primary tumours and their preoperative biopsies and affected lymph nodes was shown, the amount of subjects classified as stroma-high is consistently higher in surgical specimens, as shown in Table 5.

**TABLE 5:** This table shows the confusion matrices found in literature comparing TSR in lymph node with TSR in primary tumour and TSR in preoperative biospy with TSR in primary tumour. Ubink et al. is excluded from this table, due to a lack of exact type I and II error data (36).

Strous MTA (10)		
Lymph node	Primary tumour	
	Stroma-low (n = 107)	Stroma-high (n = 74)
stroma-low (n = 135)	92	43
stroma-high (n = 46)	15	31

Park JH (59)		
Pre-op biopsy	Primary tumour	
	Stroma-low (n = 73)	Stroma-high (n = 42)
stroma-low (n = 90)	64	26
stroma-high (n = 25)	9	16

Hansen TF (37)		
Pre-op biopsy	Primary tumour	
	Stroma-low (n = 29)	Stroma-high (n = 25)
stroma-low (n = 49)	27	22
stroma-high (n = 5)	2	3

#### 3.2.3 ROI selection methods

**FIGURE 2:**
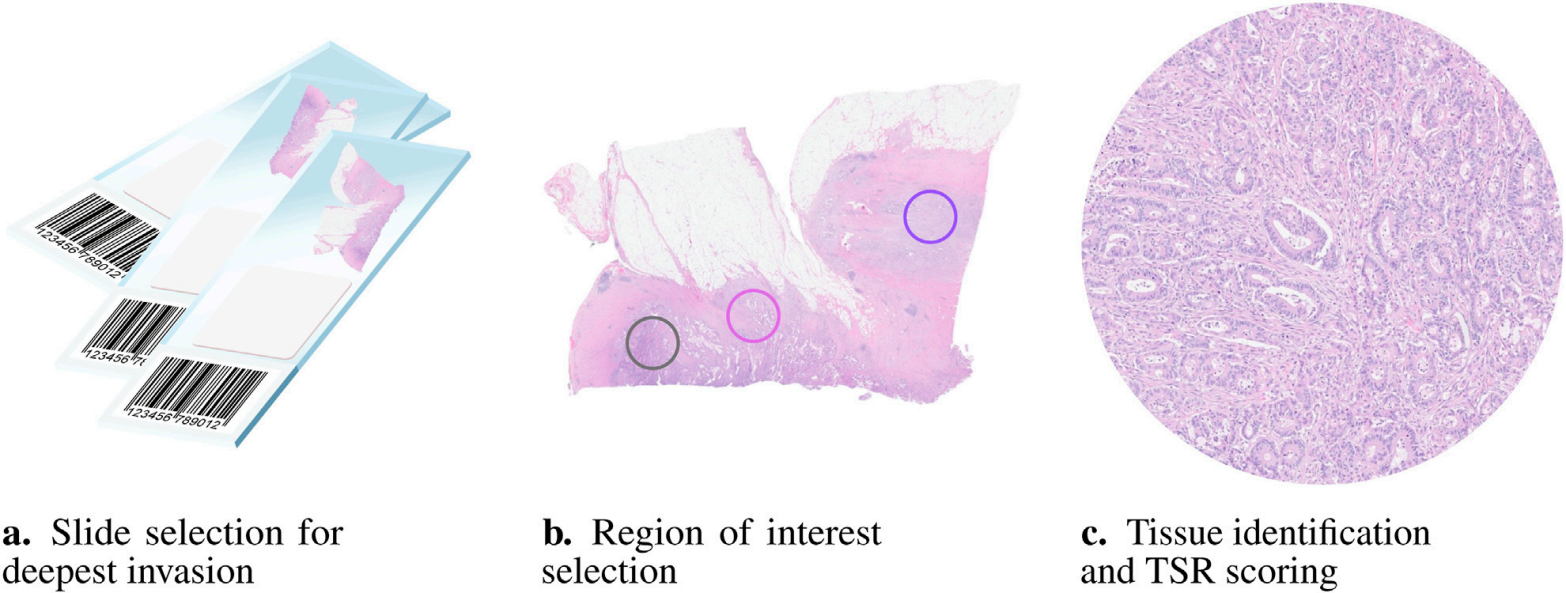
Visual representation of the pipeline used in clinical practice to manually evaluate the TSR. According to van Pelt’s 2018 recommendations (9) the first step **(a)** is selecting the slide to be scored, containing the most invasive part of the tumour. Next, **(b)** the region of interest (ROI) with the highest perceived amount of stroma is selected. Notably, tumour cells must be present at all four borders of the image field. Only one ROI is necessary, but selecting the most representative area can be challenging as multiple suitable regions may be available. Finally, **(c)** TSR is calculated after assessing the tissue types within the ROI. Lumina, necrosis, and mucin should be visually ignored for scoring, if present.

Van Pelt et al. proposed a protocol for ROI selection, which has been broadly adopted by most recent studies published after 2018 (9). The process starts by selecting slides from the most invasive part of the tumour for analysis. Initially, areas with the highest amount of stroma are identified under low magnification using a × 2.5 or × 5 lens. A single region including tumour and stromal tissue, with tumour cells present along all borders of the FOV is selected using a × 10 objective lens. In case there are multiple appropriate areas, the maximum TSR is chosen. Areas containing smooth muscle, necrotic tissue, large blood vessels, mucus, or lymphocytic aggregates should be avoided. If these features are unavoidable, they should be visually excluded during the scoring process, as seen in Figure 2.

Some studies deviated from this protocol by not requiring the presence of the tumour in the four cardinal directions of the FOV (46,50,54,60). Others calculated TSR in the infiltrative edge of tumour, the whole tumour, metastatic lesions, and tissue microarrays instead of the region with the highest perceived stromal content (19,21,40,51). Some used × 20 or × 40 objective lens, which lowers the area of the FOV (20,31,61). Four studies modified the field shape, choosing rectangular fields instead of circular ones, especially studies implementing semi-automated scoring approaches. One of these implemented an arbitrary area of 9 mm^2^. The others used an area comparable with an ocular FOV at × 10 magnification (35,49,50,62).

### 3.3 Interobserver variability

A total of 28 papers reported interobserver variability of the TSR score, as seen in Table 6. Overall the Kappa coefficient, evaluated on surgical specimen using a semi-quantitative manual approach, is widely variable between and within studies. Median scores between studies range from 0.42 to 0.876 (10,28). The largest variance between observers within the same study ranged from 0.21 to 0.90 (63).

**TABLE 6:** Interobserver variability scores of human observers.

Author	Number of observers	Number of subjects	Stroma-high subjects [%]	Tissue type	Scoring method	ICC (Range)	Kappa (Range)	Concordance (Range)
Kristensen MP (65)	2	50		SS	ROI		0.84	
Inoue H (54)	2	40	50%	SS	ROI		0.7	0.85
Kazemi A (90)	3	86	10%	SS	WSI	0.48	0.49 (0.26–0.72)	0.76 (0.72–0.78)
Firmbach D (62)	10	30	73%	SS	ROI	0.81 (0.42–0.87)	0.734	
Smit MA (72)	3	75	51%	SS	WSI	0.91 (0.89–0.94)	0.75 (0.68–0.86)	
Aboelnasr LS (31)	2	106	65%	SS	WSI		0.72	
Van de Weerd S (39)	2	83	65%	SS	WSI		0.77	
Jakab A (51)	2	185	35%	SS	ROI	0.945	0.778	
Polack M (55)	2	126	28%	LN	WSI		>0.9	0.96
Da Silva RMS (32)	2	390	53%	SS	WSI	0.823	0.746	
	2	578	27%				0.781	
Strous MTA (10)	2	201	29%	SS	WSI		0.876	
Ravensbergen CJ (45,75)	2	111		SS	WSI		0.85	
Smit MA (66)	2		44%	SS	WSI		0.83	
Liang Y (40)	2	30	78%	PB	ROI^a^	0.99		
Li T (69)	2	996	36%	SS	WSI	0.545	0.509	
Smit MA (63)	36	40	45%	SS	WSI		0.72 (0.21–0.90)	
	34						0.77 (0.51–0.97)	
	31						0.76 (0.60–0.89)	
Da Silva RMS (64)	4	98	33%–49%	SS	WSI	(0.823–0.875)	(0.673–0.813)	
Zunder SM (91)	2	33	52%	SS	WSI		0.84	
	2	69	46%				0.67	
Dang H (28)	2	183	30%	SS	WSI		0.42	
Zengin M (57)	2	172	41%	SS	WSI	0.684	0.72	
	2	172	41%			0.619	0.68	
Fu M (58)	2	353	37%	PB	WSI		0.866	
Park JH (59)	2	115	22%	SS	WSI	0.743		
Zengin M (83)	3	88	41%	SS	WSI		(0.56–0.71)	
Sandberg TP (60)	2	88		SS	WSI		0.8	
Geessink OGF (17)	2	129	33%	SS	WSI	0.736	0.578	
Huijbers A (44)	2	965	33%	SS	WSI		0.73	0.87
Eriksen AC (67)	2	50		SS	WSI		(0.70–0.75)	
Hutchins GGA (49)	2	2,975		SS	ROI^b^		0.986	0.991

SS: surgical specimen LN: lymph node, PB: pre-operative biopsy, WSI: TSR, evaluation included ROI, selection and TSR, estimation, ROI: TSR, evaluation entailed TSR, estimation in a given ROI.

^a^ Comparison of manual segmentations.

^b^ Comparison of stereology point classification.

Two tasks can be separated in the scoring of the TSR, each of which independently contribute to the reliability of the score: 1) The assignment of the optimal TSR location, and 2) the estimation of the score in that region, depicted in Figure 2. A semi-automated approach, in which only the latter of these tasks is automated does not seem to improve the Kappa score compared to the rest of literature (54). Estimating the TSR visually in a predefined ROI, also does not seem to result in an improvement of the observer variability (62). Changing the semi-quantitative task of visual eyeballing to a fully quantitative approach, such as manual tissue segmentation, and stereology analysis, appears to drastically increase the interobserver variability. Respectively these methods resulted in an ICC of 0.99 and Kappa score of 0.986 (40,49). Studies that investigated the scoring of TSR in lymph node and pre-operative biopsy, showed above average reliability, with a Kappa score of 0.866 and >0.9 (55,58). Pathologist experience and seniority also seemed to be correlated with higher interobserver reliability (64).

In order to increase the consistency of TSR scoring, an e-learning was created by Smit et al. (63). A significant improvement of reliability to the ground truth was observed after training (pre κ = 0.72 – post κ = 0.77, p = 0.002). This improvement did not fade after the washout period of 2 months (pre κ = 0.77 – post κ = 0.76, p = 0.30). This study evaluated the consistency of TSR hotspot placement for scoring. The authors noted that the spread seemed to be lower in low-stroma lesions compared to high-stroma lesions. They also found that after training, the spread in hotspot placement decreased (63).

The intraobserver TSR scoring variability was generally lower and had a smaller range compared to interobserver variability. Median Kappa scores for intraboserver variability between papers ranged from 0.77 to 0.89 (10,63,65–67).

### 3.4 Automated approaches

Automating the TSR calculation involves replacing one or more steps traditionally performed manually by pathologists, including the selection of ROIs, identification of tissue types, and calculation of the final TSR score. Included studies, focused on automating TSR scoring, can be divided into two types: semi-automated and fully automated approaches. Fourteen papers adopted semi-automated methods, where the tissue identification and the TSR estimation were performed automatically within a predefined ROI selected by pathologists (17,24,26,30,33,35,46,50–52,54,62,68,69). Five papers focused on fully automating the TSR evaluation process (47,53,56,70,71). A single study used semi-automated and fully automated pipelines to quantify the TSR (72). Most studies that developed a fully automated pipeline adopted an approach that can be roughly divided into 3 steps: (1) extracting image patches, (2) using a CNN model to classify the extracted patches into 3 or more classes, and (3) calculating TSR (47,53,70).

#### 3.4.1 ROI selection

Four out of six papers that developed a fully-automated process scored TSR on the entire tumour bulk (47,53,70,71). The other two papers focused on selecting a circular ROI, replicating the manual procedure performed by pathologists. After segmenting the WSI, the entire tumour bulk is filtered with a virtual FOV with a diameter of 1.0, 1.5 or 2.0 mm, which calculates the TSR for each possible hotspot and generates a TSR heatmap. The top k areas with the highest stroma percentages were selected as most feasible ROIs (56,72).

#### 3.4.2 Tissue identification

For automated tissue type detection, traditional methods often rely on threshold-based techniques to separate tumour and stroma tissue. These methods are typically followed by post-processing algorithms, such as morphological operations, to refine the output (24,33,54,69). While these methods can be effective for simple cases, they are limited to segmenting two tissue types and struggle to distinguish more complex features. ML methods were proposed, that classify tissue data into malignant classes: 1) tumour, 2) tumour stroma, and benign classes: 1) adipose, 2) mucinous, 3) necrotic, 4) muscular, 5) lymphatic, 6) background, and 7) healthy glandular tissue. Features extracted from super-pixels or patches are fed into a random forest or support vector machine classifier and one of the aforementioned tissues is predicted (50). Convolutional neural network (CNN) based models were used to automatically extract features from an image. Architectures such as VGG19, AlexNet, Googlenet, ResNet50 and custom models were used for patch classification (47,53,68,70,71). UNet and other fully convolutional networks were used for pixel-wise classification (17,51,52,56,62,72). Techniques like CycleGAN and transfer learning had further addressed challenges such as limited data availability and improved model performance and its ability to generalise (70,72).

#### 3.4.3 TSR validation

Validation of the automated pipeline can be performed on two levels: 1) tissue identification performance, which is reflected in the model’s ability to classify or segment different tissue types, and 2) TSR value estimation, by comparing the TSR values generated by artificial intelligence (AI) with expert assessments. CNN models have shown strong performance in tissue identification. For classification tasks, patch-wise accuracy ranges from 86.6% to 97.5%, while random forest classifiers achieve an accuracy of 76%–83%. For semantic segmentation, pixel-wise accuracy varied between 72.4% and 94.6%, Table 7. The performance metrics reported in these studies highlight the effectiveness of AI models to accurately differentiate tissue types. Firmbach et al. proposed a survey that involved expert ratings of the segmentation maps generated by the model on a scale from 1 to 10. Automated segmentations were considered high quality if the pathologists rated it ≥9/10 . This test showed that, on average, in regions with high segmentation quality, AI TSR values were 11.1–11.5 percentage points lower than those estimated by human observers, which was interpreted as human overestimation. Conversely, in cases with poor segmentation quality, the discrepancies were attributed to AI errors, particularly in challenging cases like rare tumour subtypes (62).

**TABLE 7:** Studies which provided the comparison between their automated quantification approach and the manual counterpart. The “Stroma hotspot” refers to the region with the highest perceived stroma as defined by Van Pelt et al. (9) TB: Tumour bulk, SH: Stroma hotspot, IF: Infiltrative edge, Cls: classification task, Seg: segmentation task.

Author	Tumour Region	Mode of automation	Model type	Task	Kappa	Pearson correlation	Spearman correlation	ICC	Patch/Pixel-wise accuracy
Petäinen L (70)	TB	Fully	CNN	Cls	0.33	0.57			96.1%
Firmbach D (62)		Semi	CNN	Seg		0.540			86.7%
Smit MA (72)	SH	Semi	CNN	Seg			0.88	0.78	
		Fully					0.72–0.77		
Jakab A (51)	SH	Semi	CNN		0.472			0.759	72.4%–80.0%
	IF				0.456			0.710	
	TB			Seg	0.349			0.625	
Broad A (71)	TB	Fully	CNN	Cls					86.6%
Jin HY (46)	TB	Semi	Random forest	Cls		0.865			
Li T (69)	SH	Semi	Thresholding	Seg	0.813			0.822	
Write AI (50)	SH	Semi	Random forest	Cls					76%–83%
Zhao K (53)	TB	Fully	CNN	Cls		0.939^a^		0.937	95.7%–97.5%
Geessink OGF (17)	SH	Semi	CNN	Seg	0.239			0.411–0.475	94.6%

^a^ Compared a sliding window assification to manual annotation of an ROI.

Based on the reviewed studies, the agreement between manual and automated TSR was fair to moderate for CNN models, as seen in Table 7. In these studies ICC values ranged from 0.411 to 0.937, while Kappa score values ranged between 0.239 and 0.472, indicating that automated ML-based tools are a promising method for scoring TSR, but still require further validation by experts. In addition to ML-based models, one study reported almost perfect agreement with an ICC of 0.822 and a Kappa score of 0.813, using a thresholding method (69). Smit MA et al. further compared the semi-automated method with the fully automated approach, which demonstrated good agreement, with Spearman correlation coefficients ranging from 0.76 to 0.83 (72).

## 4 Discussion

### 4.1 Usability and scoring

The TSR was shown to be a strong prognostic indicator for DFS. Most studies have also identified TSR as a statistically significant prognostic indicator for OS. While some studies did not reach statistical significance, most still showed a trend linking higher stroma content to shorter OS.

It is repeatedly mentioned in literature that TSR might be used to predict therapy resistance (10,18,40). Evidence for this is fairly scarce and included studies, investigating therapy resistance, have varying methods of TSR scoring and specific treatment received, with mixed and contradictory results (19,45). This highlights the need for further research on therapy resistance and its relation to stromal content.

This review emphasises the wide variety of TSR scoring protocols. The deviations from the protocol are most apparent in ROI location, ROI area, histological specimen type and the management of non-tumour epithelium and non-stromal tissues. This variety creates large differences in TSR evaluation, due to stromal heterogeneity of CRC (56). Stromal heterogeneity also influences the placement of the scoring ROI, which is especially apparent in stroma-high tumours, where stromal heterogeneity seems typically higher. Theoretically restricting ROI placement to a specific area of the slide, such as the infiltrative edge, could improve consistency by reducing variability in selection compared to placing ROIs across the entire slide. The TSR score calculated over this region however is consistently lower than the TSR in the hotspot suggested by Van Pelt et al. The deviation of TSR between protocols, impedes the reliable comparison of study results. Opting for a different quantification protocol, should be met with a specifically optimised cut-off value.

### 4.2 Terminology and bias

The term “tumour stroma ratio” implies the calculation of a ratio, which is mathematically defined as $\frac{\alpha }{\beta }$ , where in fact it is a percentage, defined as $\frac{\alpha }{\alpha +\beta }\;*\;100\%$ . The commonly used alternative term “tumour stroma percentage” is more in line with the stromal percentages typically used in practice to indicate stromal content. Moreover, the “tumour stroma ratio” can be misinterpreted as the ratio of tumoural stroma instead of the “ratio” of tumour to stroma. Misinterpretation of the term TSR has led to many articles defining TSR-high as stroma-high, thus leading to inconsistencies in the classification of tumours and miscommunication in research findings (10,17,19,20,23,29,47,55,64,70,73,74).

### 4.3 Scoring variability

We show that there is a wide range of reported interobserver reliability scores. On the low end Li et al. and Dang et al., reported a Kappa score of 0.51 over 996 samples and 0.42 over 183 samples respectively (28,69). On the higher end, Strous et al. and Ravensbergen et al., reported Kappa scores of 0.88 over 201 samples and 0.85 over 111 samples, respectively (10,75).

The improvement of interobserver variability after specific training for TSR scoring, as shown by the e-learning investigations of Smit et al. as well as the correlation of experience and interobserver variability as displayed by Souza da Silva et al. emphasize the need for training to improve concordance both in a research and clinical setup (63,64). Incorporating training into the multicentre studies as performed by Polack et al in the UNITED studies, greatly improves robustness of the study outcomes, and is recommended for future studies on the TSR score (18).

The median percentage of stroma-high subjects of all included papers is 37.3% ranging from 2.1% to 85.9%. This is concordant with the mean percentage of stroma-high subjects identified in the meta-analysis by Pyo et al. of 35.3% (15). The wide reported TSR range can be attributed to the inclusion and exclusion criteria of the study subjects, TSR scoring protocol, and interobserver variability. Though the reported Kappa scores indicate a moderate to substantial agreement, this should not be confused with discrepancies being acceptable for clinical adoption, Table 6. In the study by Souza da Silva et al., though the Kappa score of 0.746 indicates a substantial agreement, large discrepancies in the classification were observed. A senior pathologist classified 32.7% of samples as stroma-high whereas the baseline pathologist classified 44.9% of subjects as such, which is an increase of 37%. This shows that while Kappa can suggest substantial agreement, clinically relevant discrepancies can still occur.

The major components of variability in TSR scoring are: 1) the placement of the ROI, and 2) the estimation of the TSR percentage. Smit et al. looked at the spread of ROI placement in a set of 31–36 observers. Using a visual estimation, they found that agreeing upon a hotspot is more difficult in stroma-high cases compared to stroma-low cases. Particularly difficult cases are those including mucin lakes, large regions of necrosis, and regions were smooth muscle and stroma intermingle (63). Although it is common knowledge in TSR scoring that disagreement on ROI location is high, there is no existing metric used in literature to evaluate the placement of ROIs. For the estimation of the TSR percentage in a predefined FOV manual tissue segmentation is an almost perfect ground truth (40).

The placement of ROI is often seen as the cause for interobserver variability of the TSR score. It appears however that scoring the TSR on a predefined ROI over an independently selected ROI did not result in a measurably lower interobserver variability. Various studies have suggested that human observers face challenges in accurately estimating TSR visually. This is shown by measuring the difference of a TSR score calculated from tissue segmentation, against a TSR score visually estimated from that same region. Firmbach et al. found a mean overestimation of the TSR score of approximately 11.1%, ranging from −20% to 40% difference of the visual scoring to a quantitative baseline (62). The unreliability is also emphasised when comparing the interobserver variability of manual quantitative tasks, with the interobserver variability in semi-quantitative tasks, where a rise in variability is observed in semi-quantitative scoring (40,49). Manual quantitative measures, however, are undesirable in clinical practice, as scoring TSR using stereology is an oversimplification of the TSR and manual segmentation of tissues is tedious and time-consuming.

Overall, the interobserver variability in pre-operative biopsies and lymph nodes was lower than in surgical specimen, Table 6. In biopsies, subjects are consistently more likely to be classified as stroma-low compared to their primary tumour counterpart. It is hypothesised that this is caused by a sampling bias. The biopsy might not be performed at the level of deepest tumour invasion, and the region with the highest stroma might not be included in the biopsy. Therefore the TSR in pre-operative biopsies are a low sensitivity prognostic tool and a poor predictor for the TSR score in the primary tumour. Despite this, the TSR in pre-operative biopsy was still shown to be an independent predictor for poor prognosis (30,57–59).

### 4.4 Automated quantification

Carvalho et al. as well as Geessink et al. show that the manual TSR and automated TSR score are not comparable, and thus are currently not interchangeable (17,56). Despite this, automation of TSR quantification, using deterministic models, reduces the subjectivity of the score. More consistent and stringent adherence to the TSR scoring protocol and with it minimisation of the interobserver variability could be achieved by introduction of automated TSR scoring. On the lowest end of complexity, binary threshold methods have been used, which showed great promise to mimic human observers, with a Kappa value of 0.813 (69). However, a two-tissue segmentation model makes it impossible to adhere to the rules of tissue exclusion for quantification of the TSR. ML models are used to perform classification of tissue coordinates or patches. These methods enable the exclusion of irrelevant tissues in TSR quantification. A downside of classification methods is a lowered resolution of tissue detection introduced by patch or point sampling. Semantic segmentation overcomes this issue with a classification on pixel level. This benefit comes with the downside of having the highest model complexity, as well as the most tedious ground truth labelling process, making the acquisition of it labour intensive and the public availability of it scarce. Models performing a classification task, might not provide the resolution needed for reliably quantifying the TSR, and thus tissue segmentation models could be a preferred solution.

The largest hurdle for the use of any automated solution in histopathology is the smooth adoption of the AI model in the pathologist’s workflow. The tool should provide the pathologist with fast, human interpretable, and most importantly accurate feedback (76). A reason to favour image classification models over pixel-wise models is the simpler ground-truth labelling and architecture complexity, which reduces response time in clinical setting and makes them cheaper to train. Besides this, the image classification task generalizes better with a low amount of data compared to segmentation models. However, their spatial resolution cannot offer a fine-grained human interpretable response, nor is it accurate enough for finding the TSR score. The high resolution output of pixel-wise models are precise and can provide human interpretable feedback (77). These segmentation models require more processing time, which adds complexity to the integration of these solutions into clinical workflow (78). In TSR specifically, the greatest hurdle is the discordance between expert estimation of the TSR and automatically evaluated TSR scores, despite models’ high performance for the identification of tissues (17,62,70).

The common approach for fully automated methods found in literature, is creating an AI classification model for benign and malignant tissue types that performs the classification of all patches containing tissue within the WSI, and calculates the TSR for the entire tumour bulk (53,70,71). Petäinen et al. report a Kappa score of 0.33, despite a patch classification accuracy of 96.1%. This discrepancy is caused by comparing an automated quantification of whole tumour TSR with a semi-quantitative quantification of the highest perceived stroma region. This discrepancy disappears when comparing automated and manual approaches for identical quantification strategies (53). Two studies performed a fully automated TSR quantification of the highest perceived stroma region and compared its performance to human observers (56). This translated to a strong Pearson correlation between manual and automated quantification (72).

Note that the optimal cut-off for prediction of prognosis was calculated using scores generated semi-quantitively, using a conventional microscope. The TSR is systematically underestimated by human observers, which leads to a larger amount of subjects being classified as stroma-high in automated quantification (17,51,62,69). This means that for effective stratification of subjects using automated TSR a new optimal cut-off may need to be defined.

Evaluating a fully automated approach requires assessing each step independently to determine its specific contribution and identify sources of inaccuracies. Based on the reviewed studies, only one paper has conducted such assessments for TSR estimation (62). Despite this finding, most studies in the literature assessed TSR estimation by comparing automated TSR-score with manual TSR-score. However, manual estimation remains subjective, making the ground-truth of the comparison debatable. Additionally, to date, no studies have evaluated ROI selection in fully automated TSR quantification pipelines. This evaluation is highly subjective, as its current gold standard is consensus of expert judgement. It is important to note that defining a quantitative metric for evaluating hotspot selection is challenging. Multiple adequate ROIs exist within a slide, making commonly used metrics such as Dice or Euclidean distance unfeasible.

The systematic overestimation of the TSR score can explain the strongly improved kappa scores, for a median cut-off compared to a 50% cut-off in the automated tool proposed by Geessink et al. ( κ=0.239 to κ=0.521 ) (17). It could also explain the optimal clinical threshold of 80% that was found in the study performed by Carvalho et al. (56). In general, upon evaluating the automated scoring methods, we observe that the Kappa score for classification agreement are poor. Metrics evaluating a monotonic or linear relationship, tend to be higher, solidifying the findings by Carvalho et al. and Geessink et al., Table 7.

Despite these findings there have been no attempts to isolate the cause of this systematic overestimation yet. To identify the root cause of the discrepancy, we propose to evaluate the effectors to the TSR score individually, both for the manual and automated process. In the automated process, these are: 1) tissue identification, 2) automated region selection, and 3) TSR evaluation, which is the tool’s capability of translating identified tissues to a percentage score. In the manual process, these are: 1) The ability to find an optimal ROI, and 2) the ability to accurately eyeball the TSR score in a given region. To our knowledge, this last factor has not yet been addressed. We suggest a study setup in which pathologists eyeball the TSR score on previously fully annotated tumour regions after a defined washout period. This would quantify the systematic error of pathologists when visually estimating an ROI. Besides this, we strongly advise to make use of the “discrepancy ratio” in the evaluation of automated solutions in the field of pathology, as it is specifically designed to evaluate the performance of automated tools in tasks where there is frequent disagreement between experts (79).

Some limitations of this scoping review are that it included only articles after 2018, which excludes early research and thus could exclude articles that have led to the creation of the standardised scoring protocol (9). This might obscure the rationale for specific steps in these recommendations. Besides this, conference papers were excluded from this review, which might result in an underestimation of the number of automated TSR pipelines developed.

Also, this review focused solely on papers related to colorectal cancer, which may have limited the identification of additional rules and techniques for TSR calculation, such as the formula applied and the ROI selection process, as well as automated techniques for TSR scoring.

An increase in published articles can be seen as a trend over time, with a mean of 10 articles per year published from 2018 to 2020 and a mean of 15 articles published per year from 2021 to 2025. Interestingly, most of existing literature is dominated by a few research groups. Before 2021 the three most prominent groups were responsible for 64% of published studies. Averaged over all included studies in this review, these research groups are responsible for 38% of all published studies. The single most dominant research group is responsible for 22% of published studies from 2018 onwards. A reader unaware of this imbalance in publishing might form an incomplete view of the existing evidence and clinical practices.

## 5 Conclusion

TSR is a robust indicator for DFS and OS, and can possibly predict therapy resistance. Adoption of a single procedure for TSR scoring is inconsistent across research communities. This is most apparent in ROI selection and determining the cut-off value for risk stratification. TSR scoring in pre-operative biopsies may be a significant indicator for poor prognosis, despite being a poor predictor for TSR in the primary tumour. The scoring procedure followed is strongly correlated with the optimal cut-off for stratifying subjects into risk categories, which is likely caused by stromal heterogeneity of colorectal lesions. Additionally, it influences the inter-observer variability. Kappa scores for manual semi-quantitative scoring solutions range from 0.42 to 0.88. Automated scoring solutions are proposed to reduce labour and increase interobserver reliability. Despite showing high model performance, comparisons between manual and automated TSR scores result in kappa scores ranging from 0.239 to 0.472. In order to adopt TSR scoring in clinical practice, it is essential to standardise the scoring process, including the equation, region of interest selection, and cut-off value. Moreover, the development of an automated tool to assist pathologists requires a well-defined validation process that goes beyond comparisons with human observers and incorporates additional methods to assess the tool’s accuracy, reliability and clinical usability.

## Data Availability

The original contributions presented in the study are included in the article/Supplementary Material, further inquiries can be directed to the corresponding author.

## Abbreviations

AI, Artificial Intelligence; CC, Colon Carcinoma; CNN, Convolutional Neural Network; CRC, Colorectal Carcinoma; CSS, Cancer Specific Survival; DFS, Disease Free Survival; FOV, Field of View; ICC, Intra-class correlation; OS, Overall Survival; RC, Rectal Carcinoma; ROI, Region of Interest; SP, Stroma Proportion; TP, Tumour Proportion; TSP, Tumour Stroma Percentage; TSR, Tumour Stroma Ratio.
