# Supplementary material for: A scoping review of TSR analysis in colorectal cancer: implications for automated solutions
Source: Oncol Rev. 2025 Oct 28;19:1605383. doi: 10.3389/or.2025.1605383 (PMC12603809; doi:10.3389/or.2025.1605383)
Supplement: Supplementary file 1 [file DataSheet1.PDF]

Supplementary Material

1 SUPPLEMENTARY TABLES

Manual stroma content scoring

Author	Year	Number of subjects	Stroma-high subjects [%]	Disease	Tumour stages
Kristensen MP (1)	2025	497	31%	CC	II
Pujani M (2)	2024	65	45%	CRC	I-III
Unal Kocabey D (3)	2024	126	49%	CRC	I-IV
Hong SA (4)	2024	323	33%	CRC	I-IV
Li B (5)	2024	106	43%	CRC	I-IV
Fekete Z† (6)	2024	74	7%	CRC	II-III
Jakab A (7)	2024	185	35%	CRC	I-IV
Polack M (8)	2024	1388	31%	CC	II-III
Magnusson MI (9)	2023	2162	26%	CRC	I-IV
Tian W (10)	2023	153	41%	RC	II-III
Khan AA (11)	2023	40	65%	CRC	I-III
Kazemi A (12)	2023			CRC	II-III
Pyo JS‡ (13)	2023	2999	35%	CRC	I-IV
Strous MTA (14)	2023	201	29%	CC	I-II
Aboelnasr LS (15)	2023	103	65%	CRC	I-IV
Van de Weerd S (16)	2022	218	33%	CC	II-III
Wang Q (17)	2022	114<br>60	41%<br>40%	CRC	I-III
Loft MK (18)	2022	86	9%	RC	0-I
Polack M (19)	2022	126<br>(lymph nodes)	38%	CRC	III
Hu S (20)	2022	66	62%	RC	I-III
Yim K (21)	2022	85		CRC	I-IV
Strous MTA (22)	2022	578<br>201	27%<br>29%	CC	I-III
Sullivan L† (23)	2022				
Fan S (24)	2022	207	45%	CRC	II
Strous MTA (25)	2022	187	41%	RC	I-III
Souza da Silva RM (26)	2022	390	53%	CRC	I-IV
Ravensbergen CJ (27)	2021	333<br>229	39%<br>50%	CC	I-IV
Smit MA (28)	2021	246	44%	CC	II-III
Gao J‡ (29)	2021	4857	30%	CRC	I-III
Ravensbergen CJ (30)	2021	366	40%	CC	I-IV
Liang Y (31)	2021	248	78%	RC	II-III
Zhu Y‡ (32)	2021	5408	32%	CRC	I-IV
Cai C (33)	2021	149	43%	RC	I-III

Kang G (34)	2021	266	30%	CRC	I-IV
Smit MA (35)	2021	77	45%	CC	II-III
Souza da Silva RM (36)	2021	98	33%–49%	CRC	I-IV
Zhang Y (37)	2021	147	12%	CRC	I-IV
Zunder SM (38)	2020	33<br>69	52%<br>46%	RC	I-IV<br>II-III
Dang H (39)	2020	223	30%	CRC	I
Zengin M (40)	2020	172	41%	CC	III-IV
Fu M (41)	2020	353<br>177	38%<br>37%	CRC	I-III
Park JH (42)	2020	115	22%	CRC	I-III
Van Wyk HC (43)	2019	952	25%	CRC	I-IV
Zengin M (44)	2019	88	41%	CC	I
Zunder SM (45)	2019	1103	30%	CC	II-III
Den Uil (46)	2019	107	48%	CC	II-III
Sandberg TP (47)	2019	201	48%	CRC	I-IV
Van Pelt† (48)	2018			CC	
Eriksen AC (49)	2018	573	29%	CC	II
Van Pelt† (50)	2018				
Zunder SM (51)	2018	1212	29%	CC	II-III
Huijbers A (52)	2018	965	33%	CC	II-III
Hansen TF (53)	2018	65	53%	CC	II-III
Eriksen AC (54)	2018	43		CC	II
Hutchins GGA (55)	2018	1800	75%	CRC	II-III
Ubink I (56)	2018	24	79%	CRC	IV

Automated stroma content scoring

Author	Year	Number of subjects	Stroma-high subjects [%]	Disease	Tumour stages
Carvalho R (57)	2025	1317	25%	CRC	I-IV
Zhao Y (58)	2025	179	20%	CRC	II
Zhao Q (59)	2024	302		RC	II-III
Sinicrope FA (60)	2024	380		CC	III
Inoue H (61)	2023	200	50%	CC	II-III
Petaïnen L (62)	2023	1343	37%	CRC	I-IV
Firmbach D (63)	2023	59		CC	II-III
Smit MA (64)	2023	75	51%	CC	II-III
Yang J (65)	2022	544<br>466	28%<br>55%	CRC	I-III
Jakab A (66)	2022	185	34%	CRC	I-IV
Broad A (67)	2022	689		CRC	
Jin HY (68)	2022	487	20%	CRC	III
Zhao Z (69)	2021	143<br>179<br>174	2%<br>24%<br>39%	CRC	I<br>II<br>IV

Jones HJS (70)	2021	143	8%	RC	I-IV
Miller S (71)	2021	253	35%	CC	II-III
Li T (72)	2021	996	36%	CRC	I-IV
Wright AI (73)	2021	2211		CRC	II-III
Zhao K (74)	2020	499<br>315	28%<br>51%	CRC	I-IV<br>II-III
Martin B (75)	2020	206	86%	CC	II-III
Geessink OGF (76)	2019	129	33%	RC	I-III

Table S1: Study specifications of all included articles. †These review articles are used for understanding of general concepts and identification of knowledge gaps.
‡These studies have performed a meta-analysis for TSR as prognostic indicator for CRC.

KM Grade<br>0 & 1 – 2 & 3	TSR<br>$\leq$ 50% – >50% stroma	GMS<br>0 – 1 – 2
high	low/high	0
low	low	1
low	high	2

Table S2: Classification of the GMS according to Jakab et al. (7)

REFERENCES

1 .Kristensen MP, Korsgaard U, Timm S, Hansen TF, Zlobec I, Hager H, et al. engPrognostic value of tumor-stroma ratio in a screened stage II colon cancer population: intratumoral site-specific assessment and tumor budding synergy. *Modern Pathology: An Official Journal of the United States and Canadian Academy of Pathology, Inc* (2025) 100738. doi:10.1016/j.modpat.2025.100738.

2 .Pujani M, Singh K, Agarwal C, Chauhan V, Prasad S, Singh M, et al. enPrognostic Role of Tumor-Infiltrating Lymphocytes, Tumor Budding, Tumor Border Configuration, and Tumor Stroma Ratio in Colorectal Carcinoma. *Indian Journal of Surgical Oncology* (2024). doi:10.1007/s13193-024-02127-1.

3 .Unal Kocabey D, Cakir IE. engThe prognostic significance of growth pattern, tumor budding, poorly differentiated clusters, desmoplastic reaction pattern and tumor-stroma ratio in colorectal cancer and an evaluation of their relationship with KRAS, NRAS, BRAF mutations. *Annals of Diagnostic Pathology* **73** (2024) 152375. doi:10.1016/j.anndiagpath.2024.152375.

4 .Hong SA, Lee HJ, Kim OH, Hong M, Kim JW, Kim JY. engMicroRNA-206 overexpression is associated with a prominent inflammatory reaction and a favorable colorectal cancer prognosis. *Pathology, Research and Practice* **263** (2024) 155573. doi:10.1016/j.prp.2024.155573.

5 .Li B, Chen L, Huang Y, Wu M, Fang W, Zou X, et al. engAre the tumor microenvironment characteristics of pretreatment biopsy specimens of colorectal cancer really effectively predict the efficacy of neoadjuvant therapy: A retrospective multicenter study. *Medicine* **103** (2024) e39429. doi:10.1097/MD.0000000000039429.

6 .Fekete Z, Ignat P, Resiga AC, Todor N, Muntean AS, Resiga L, et al. engUnselective Measurement of Tumor-to-Stroma Proportion in Colon Cancer at the Invasion Front-An Elusive Prognostic Factor:

Original Patient Data and Review of the Literature. *Diagnostics (Basel, Switzerland)* **14** (2024) 836. doi:10.3390/diagnostics14080836.

7 .Jakab A, Patai , Darvas M, Tormássi-Bély K, Micsik T. engMicroenvironment, systemic inflammatory response and tumor markers considering consensus molecular subtypes of colorectal cancer. *Pathology oncology research: POR* **30** (2024) 1611574. doi:10.3389/pore.2024.1611574.

8 .Polack M, Smit MA, van Pelt GW, Roodvoets AGH, Meershoek-Klein Kranenbarg E, Putter H, et al. engResults from the UNITED study: a multicenter study validating the prognostic effect of the tumor-stroma ratio in colon cancer. *ESMO open* **9** (2024) 102988. doi:10.1016/j.esmoop.2024.102988.

9 .Magnusson MI, Agnarsson BA, Jonasson JG, Tryggvason T, Aeffner F, le Roux L, et al. engHistopathology and levels of proteins in plasma associate with survival after colorectal cancer diagnosis. *British Journal of Cancer* **129** (2023) 1142–1151. doi:10.1038/s41416-023-02374-z.

10 .Tian W, Yang Y, Qin Q, Zhang L, Wang Z, Su L, et al. engVimentin and tumor-stroma ratio for neoadjuvant chemoradiotherapy response prediction in locally advanced rectal cancer. *Cancer Science* **114** (2023) 619–629. doi:10.1111/cas.15610.

11 .Khan AA, Malik S, Jacob S, Aden D, Ahuja S, Zaheer S, et al. engPrognostic evaluation of cancer associated fibrosis and tumor budding in colorectal cancer. *Pathology, Research and Practice* **248** (2023) 154587. doi:10.1016/j.prp.2023.154587.

12 .Kazemi A, Gharib M, Mohamadian Roshan N, Taraz Jamshidi S, Stögbauer F, Eslami S, et al. engAssessment of the Tumor-Stroma Ratio and Tumor-Infiltrating Lymphocytes in Colorectal Cancer: Inter-Observer Agreement Evaluation. *Diagnostics (Basel, Switzerland)* **13** (2023) 2339. doi: 10.3390/diagnostics13142339.

13 .Pyo JS, Kim NY, Min KW, Kang DW. engSignificance of Tumor-Stroma Ratio (TSR) in Predicting Outcomes of Malignant Tumors. *Medicina (Kaunas, Lithuania)* **59** (2023) 1258. doi:10.3390/medicina59071258.

14 .Strous MTA, van der Linden RLA, Gubbels ALHM, Faes TKE, Bosscha K, Bronkhorst CM, et al. engNode-negative colon cancer: histological, molecular, and stromal features predicting disease recurrence. *Molecular Medicine (Cambridge, Mass.)* **29** (2023) 77. doi:10.1186/s10020-023-00677-8.

15 .Aboelnasr LS, El-Rebey HS, Mohamed A, Abdou AG. engThe Prognostic Impact of Tumor Border Configuration, Tumor Budding and Tumor Stroma Ratio in Colorectal Carcinoma. *Turk Patoloji Dergisi* **39** (2023) 83–93. doi:10.5146/tjpath.2022.01579.

16 .van de Weerd S, Smit MA, Roelands J, Mesker WE, Bedognetti D, Kuppen PJK, et al. engCorrelation of Immunological and Histopathological Features with Gene Expression-Based Classifiers in Colon Cancer Patients. *International Journal of Molecular Sciences* **23** (2022) 12707. doi:10.3390/ijms232012707.

17 .Wang Q, Shen X, An R, Bai J, Dong J, Cai H, et al. engPeritumoral tertiary lymphoid structure and tumor stroma percentage predict the prognosis of patients with non-metastatic colorectal cancer. *Frontiers in Immunology* **13** (2022) 962056. doi:10.3389/fimmu.2022.962056.

18 .Loft MK, Pedersen MRV, Lindebjerg J, Rahr HB, Rafaelsen SR. engEndorectal Ultrasound Shear-Wave Elastography of Complex Rectal Adenoma and Early Rectal Cancer. *Diagnostics (Basel, Switzerland)* **12** (2022) 2166. doi:10.3390/diagnostics12092166.

19 .Polack M, Hagenaars SC, Couwenberg A, Kool W, Tollenaar RAEM, Vogel WV, et al. engCharacteristics of tumour stroma in regional lymph node metastases in colorectal cancer patients: a theoretical framework for future diagnostic imaging with FAPI PET/CT. *Clinical & Translational Oncology: Official Publication of the Federation of Spanish Oncology Societies and of the National Cancer Institute of Mexico* **24** (2022) 1776–1784. doi:10.1007/s12094-022-02832-9.

20 .Hu S, Xing X, Liu J, Liu X, Li J, Jin W, et al. engCorrelation between apparent diffusion coefficient and tumor-stroma ratio in hybrid 18F-FDG PET/MRI: preliminary results of a rectal cancer cohort study. *Quantitative Imaging in Medicine and Surgery* **12** (2022) 4213–4225. doi:10.21037/qims-21-938.

21 .Yim K, Jang WM, Cho U, Sun DS, Chong Y, Seo KJ. engIntratumoral Budding in Pretreatment Biopsies, among Tumor Microenvironmental Components, Can Predict Prognosis and Neoadjuvant Therapy Response in Colorectal Adenocarcinoma. *Medicina (Kaunas, Lithuania)* **58** (2022) 926. doi:10.3390/medicina58070926.

22 .Strous MTA, Faes TKE, Gubbels ALHM, van der Linden RLA, Mesker WE, Bosscha K, et al. engA high tumour-stroma ratio (TSR) in colon tumours and its metastatic lymph nodes predicts poor cancer-free survival and chemo resistance. *Clinical & Translational Oncology: Official Publication of the Federation of Spanish Oncology Societies and of the National Cancer Institute of Mexico* **24** (2022) 1047–1058. doi:10.1007/s12094-021-02746-y.

23 .Sullivan L, Pacheco RR, Kmeid M, Chen A, Lee H. engTumor Stroma Ratio and Its Significance in Locally Advanced Colorectal Cancer. *Current Oncology (Toronto, Ont.)* **29** (2022) 3232–3241. doi:10.3390/curroncol29050263.

24 .Fan S, Cui X, Zheng L, Ma W, Zheng S, Wang J, et al. engPrognostic value of desmoplastic stromal reaction, tumor budding and tumor-stroma ratio in stage II colorectal cancer. *Journal of Gastrointestinal Oncology* **13** (2022) 2903–2921. doi:10.21037/jgo-22-758.

25 .Strous MTA, Faes TKE, Heemskerk J, Lohman BGPM, Simons PCG, Janssen Heijnen MLG, et al. engTumour-stroma ratio to predict pathological response to neo-adjuvant treatment in rectal cancer. *Surgical Oncology* **45** (2022) 101862. doi:10.1016/j.suronc.2022.101862.

26 .Souza D, Queiroga E, De T, Cunha K, Dias E. enStromal scoring in advanced colon and rectal cancer: Stroma-rich tumors and their association with aggressive phenotypes. *Archive of Oncology* **28** (2022) 1–6. doi:10.2298/AOO210403003S.

27 .Ravensbergen CJ, Kuruc M, Polack M, Crobach S, Putter H, Gelderblom H, et al. engThe Stroma Liquid Biopsy Panel Contains a Stromal-Epithelial Gene Signature Ratio That Is Associated with the Histologic Tumor-Stroma Ratio and Predicts Survival in Colon Cancer. *Cancers* **14** (2021) 163. doi:10.3390/cancers14010163.

28 .Smit MA, van Pelt GW, Terpstra V, Putter H, Tollenaar RAEM, Mesker WE, et al. engTumour-stroma ratio outperforms tumour budding as biomarker in colon cancer: a cohort study. *International Journal of Colorectal Disease* **36** (2021) 2729–2737. doi:10.1007/s00384-021-04023-4.

29 .Gao J, Shen Z, Deng Z, Mei L. engImpact of Tumor-Stroma Ratio on the Prognosis of Colorectal Cancer: A Systematic Review. *Frontiers in Oncology* **11** (2021) 738080. doi:10.3389/fonc.2021.738080.

30 .Ravensbergen CJ, Polack M, Roelands J, Crobach S, Putter H, Gelderblom H, et al. engCombined Assessment of the Tumor-Stroma Ratio and Tumor Immune Cell Infiltrate for Immune Checkpoint Inhibitor Therapy Response Prediction in Colon Cancer. *Cells* **10** (2021) 2935. doi:10.3390/cells10112935.

31 .Liang Y, Zhu Y, Lin H, Zhang S, Li S, Huang Y, et al. engThe value of the tumour-stroma ratio for predicting neoadjuvant chemoradiotherapy response in locally advanced rectal cancer: a case control study. *BMC cancer* **21** (2021) 729. doi:10.1186/s12885-021-08516-x.

32 .Zhu Y, Jin Z, Qian Y, Shen Y, Wang Z. engPrognostic Value of Tumor-Stroma Ratio in Rectal Cancer: A Systematic Review and Meta-analysis. *Frontiers in Oncology* **11** (2021) 685570. doi:10.3389/fonc.2021.685570.

33 .Cai C, Hu T, Gong J, Huang D, Liu F, Fu C, et al. engMultiparametric MRI-based radiomics signature for preoperative estimation of tumor-stroma ratio in rectal cancer. *European Radiology* **31** (2021) 3326–3335. doi:10.1007/s00330-020-07403-6.

34 .Kang G, Pyo JS, Kim NY, Kang DW. engClinicopathological Significances of Tumor-Stroma Ratio (TSR) in Colorectal Cancers: Prognostic Implication of TSR Compared to Hypoxia-Inducible Factor-1 Expression and Microvessel Density. *Current Oncology (Toronto, Ont.)* **28** (2021) 1314–1324. doi:10.3390/curroncol28020125.

35 .Smit MA, van Pelt GW, Dequeker EM, Al Dieri R, Tollenaar RA, van Krieken JHJ, et al. enge-Learning for Instruction and to Improve Reproducibility of Scoring Tumor-Stroma Ratio in Colon Carcinoma: Performance and Reproducibility Assessment in the UNITED Study. *JMIR formative research* **5** (2021) e19408. doi:10.2196/19408.

36 .Souza da Silva RM, Queiroga EM, Paz AR, Neves FFP, Cunha KS, Dias EP. engStandardized Assessment of the Tumor-Stroma Ratio in Colorectal Cancer: Interobserver Validation and Reproducibility of a Potential Prognostic Factor. *Clinical Pathology (Thousand Oaks, Ventura County, Calif.)* **14** (2021) 2632010X21989686. doi:10.1177/2632010X21989686.

37 .Zhang Y, Liu Y, Qiu X, Yan B. engConcurrent Comparison of the Prognostic Values of Tumor Budding, Tumor Stroma Ratio, Tumor Infiltrating Pattern and Lymphocyte-to-Monocyte Ratio in Colorectal Cancer Patients. *Technology in Cancer Research & Treatment* **20** (2021) 15330338211045826. doi:10.1177/15330338211045826.

38 .Zunder SM, Perez-Lopez R, de Kok BM, Raciti MV, van Pelt GW, Dienstmann R, et al. engCorrelation of the tumour-stroma ratio with diffusion weighted MRI in rectal cancer. *European Journal of Radiology* **133** (2020) 109345. doi:10.1016/j.ejrad.2020.109345.

39 .Dang H, van Pelt GW, Haasnoot KJ, Backes Y, Elias SG, Seerden TC, et al. engTumour-stroma ratio has poor prognostic value in non-pedunculated T1 colorectal cancer: A multi-centre case-cohort study. *United European Gastroenterology Journal* **9** (2020) 2050640620975324. doi:10.1177/2050640620975324.

40 .Zengin M, Benek S. engThe Proportion of Tumour-Stroma in Metastatic Lymph Nodes is An Accurately Prognostic Indicator of Poor Survival for Advanced-Stage Colon Cancers. *Pathology oncology research: POR* **26** (2020) 2755–2764. doi:10.1007/s12253-020-00877-1.

41 .Fu M, Chen D, Luo F, Li M, Wang Y, Chen J, et al. engAssociation of the tumour stroma percentage in the preoperative biopsies with lymph node metastasis in colorectal cancer. *British Journal of Cancer* **122** (2020) 388–396. doi:10.1038/s41416-019-0671-7.

42 .Park JH, van Wyk H, McMillan DC, Edwards J, Orange C, Horgan PG, et al. engPreoperative, biopsy-based assessment of the tumour microenvironment in patients with primary operable colorectal cancer. *The Journal of Pathology. Clinical Research* **6** (2020) 30–39. doi:10.1002/cjp2.143.

43 .van Wyk HC, Roseweir A, Alexander P, Park JH, Horgan PG, McMillan DC, et al. engThe Relationship Between Tumor Budding, Tumor Microenvironment, and Survival in Patients with Primary Operable Colorectal Cancer. *Annals of Surgical Oncology* **26** (2019) 4397–4404. doi:10.1245/s10434-019-07931-6.

44 .Zengin M. engTumour Budding and Tumour Stroma Ratio are Reliable Predictors for Death and Recurrence in Elderly Stage I Colon Cancer Patients. *Pathology, Research and Practice* **215** (2019) 152635. doi:10.1016/j.prp.2019.152635.

45 .Zunder S, van der Wilk P, Gelderblom H, Dekker T, Mancao C, Kiialainen A, et al. engStromal organization as predictive biomarker for the treatment of colon cancer with adjuvant bevacizumab; a

post-hoc analysis of the AVANT trial. *Cellular Oncology (Dordrecht, Netherlands)* **42** (2019) 717–725. doi:10.1007/s13402-019-00449-9.

46 .den Uil SH, van den Broek E, Coupé VMH, Vellinga TT, Delis-van Diemen PM, Bril H, et al. engPrognostic value of microvessel density in stage II and III colon cancer patients: a retrospective cohort study. *BMC gastroenterology* **19** (2019) 146. doi:10.1186/s12876-019-1063-4.

47 .Sandberg TP, Sweere I, van Pelt GW, Putter H, Vermeulen L, Kuppen PJ, et al. engPrognostic value of low CDX2 expression in colorectal cancers with a high stromal content - a short report. *Cellular Oncology (Dordrecht, Netherlands)* **42** (2019) 397–403. doi:10.1007/s13402-019-00436-0.

48 .van Pelt GW, Kjær-Frifeldt S, van Krieken JHJM, Al Dieri R, Morreau H, Tollenaar RaEM, et al. engScoring the tumor-stroma ratio in colon cancer: procedure and recommendations. *Virchows Archiv: An International Journal of Pathology* **473** (2018) 405–412. doi:10.1007/s00428-018-2408-z.

49 .Eriksen AC, Sørensen FB, Lindebjerg J, Hager H, dePont Christensen R, Kjær-Frifeldt S, et al. engThe prognostic value of tumour stroma ratio and tumour budding in stage II colon cancer. A nationwide population-based study. *International Journal of Colorectal Disease* **33** (2018) 1115–1124. doi:10.1007/s00384-018-3076-9.

50 .van Pelt GW, Sandberg TP, Morreau H, Gelderblom H, van Krieken JHJM, Tollenaar RAEM, et al. engThe tumour-stroma ratio in colon cancer: the biological role and its prognostic impact. *Histopathology* **73** (2018) 197–206. doi:10.1111/his.13489.

51 .Zunder SM, van Pelt GW, Gelderblom HJ, Mancao C, Putter H, Tollenaar RA, et al. engPredictive potential of tumour-stroma ratio on benefit from adjuvant bevacizumab in high-risk stage II and stage III colon cancer. *British Journal of Cancer* **119** (2018) 164–169. doi:10.1038/s41416-018-0083-0.

52 .Huijbers A, van Pelt GW, Kerr RS, Johnstone EC, Tollenaar RAEM, Kerr DJ, et al. engThe value of additional bevacizumab in patients with high-risk stroma-high colon cancer. A study within the QUASAR2 trial, an open-label randomized phase 3 trial. *Journal of Surgical Oncology* **117** (2018) 1043–1048. doi:10.1002/jso.24998.

53 .Hansen TF, Kjær-Frifeldt S, Lindebjerg J, Rafaelsen SR, Jensen LH, Jakobsen A, et al. engTumor-stroma ratio predicts recurrence in patients with colon cancer treated with neoadjuvant chemotherapy. *Acta Oncologica (Stockholm, Sweden)* **57** (2018) 528–533. doi:10.1080/0284186X.2017.1385841.

54 .Eriksen AC, Andersen JB, Lindebjerg J, dePont Christensen R, Hansen TF, Kjær-Frifeldt S, et al. engDoes heterogeneity matter in the estimation of tumour budding and tumour stroma ratio in colon cancer? *Diagnostic Pathology* **13** (2018) 20. doi:10.1186/s13000-018-0697-9.

55 .Hutchins GGA, Treanor D, Wright A, Handley K, Magill L, Tinkler-Hundal E, et al. engIntratumoral stromal morphometry predicts disease recurrence but not response to 5-fluorouracil-results from the QUASAR trial of colorectal cancer. *Histopathology* **72** (2018) 391–404. doi:10.1111/his.13326.

56 .Ubink I, van Eden WJ, Snaebjornsson P, Kok NFM, van Kuik J, van Grevenstein WMU, et al. engHistopathological and molecular classification of colorectal cancer and corresponding peritoneal metastases. *The British Journal of Surgery* **105** (2018) e204–e211. doi:10.1002/bjs.10788.

57 .Carvalho R, Zander T, Barroso VM, Bekisoglu A, Zerbe N, Klein S, et al. engAI-based tumor-stroma ratio quantification algorithm: comprehensive evaluation of prognostic role in primary colorectal cancer. *Virchows Archiv: An International Journal of Pathology* (2025). doi:10.1007/s00428-025-04048-y.

58 .Zhao Y, Xia S, Zhao X, Song Z, Wang F, Mao L, et al. engDNA ploidy combined with tumor stroma as a biomarker for predicting the prognosis of stage II colorectal cancer patients and identifying candidates for chemotherapy. *World Journal of Surgical Oncology* **23** (2025) 49. doi:10.1186/s12957-025-03693-6.

59 .Zhao Q, Zhong H, Guan X, Wan L, Zhao X, Zou S, et al. engRole of microenvironment characteristics and MRI radiomics in the risk stratification of distant metastases in rectal cancer: A diagnostic study. *International Journal of Surgery (London, England)* (2024). doi:10.1097/JS9.0000000000001916.

60 .Sinicrope FA, Nelson GD, Saberzadeh-Ardestani B, Segovia DI, Graham RP, Wu C, et al. engUse of Deep Learning to Evaluate Tumor Microenvironmental Features for Prediction of Colon Cancer Recurrence. *Cancer Research Communications* **4** (2024) 1344–1350. doi:10.1158/2767-9764.CRC-24-0031.

61 .Inoue H, Kudou M, Shiozaki A, Kosuga T, Shimizu H, Kiuchi J, et al. engValue of the Tumor-Stroma Ratio and Structural Heterogeneity Measured by a Novel Semiautomatic Image Analysis Technique for Predicting Survival in Patients With Colon Cancer. *Diseases of the Colon and Rectum* **66** (2023) 1449–1461. doi:10.1097/DCR.0000000000002570.

62 .Petäinen L, Väyrynen JP, Ruusuvuori P, Pölönen I, Äyrämö S, Kuopio T. engDomain-specific transfer learning in the automated scoring of tumor-stroma ratio from histopathological images of colorectal cancer. *PloS One* **18** (2023) e0286270. doi:10.1371/journal.pone.0286270.

63 .Firmbach D, Benz M, Kuritcyn P, Bruns V, Lang-Schwarz C, Stuebs FA, et al. engTumor-Stroma Ratio in Colorectal Cancer-Comparison between Human Estimation and Automated Assessment. *Cancers* **15** (2023) 2675. doi:10.3390/cancers15102675.

64 .Smit MA, Ciompi F, Bokhorst JM, van Pelt GW, Geessink OGF, Putter H, et al. engDeep learning based tumor-stroma ratio scoring in colon cancer correlates with microscopic assessment. *Journal of Pathology Informatics* **14** (2023) 100191. doi:10.1016/j.jpi.2023.100191.

65 .Yang J, Ye H, Fan X, Li Y, Wu X, Zhao M, et al. engArtificial intelligence for quantifying immune infiltrates interacting with stroma in colorectal cancer. *Journal of Translational Medicine* **20** (2022) 451. doi:10.1186/s12967-022-03666-3.

66 .Jakab A, Patai , Micsik T. engDigital image analysis provides robust tissue microenvironment-based prognosticators in patients with stage I-IV colorectal cancer. *Human Pathology* **128** (2022) 141–151. doi:10.1016/j.humpath.2022.07.003.

67 .Broad A, Wright AI, de Kamps M, Treanor D. engAttention-guided sampling for colorectal cancer analysis with digital pathology. *Journal of Pathology Informatics* **13** (2022) 100110. doi:10.1016/j.jpi.2022.100110.

68 .Jin HY, Yoo SY, Lee JA, Wen X, Kim Y, Park HE, et al. engCombinatory statuses of tumor stromal percentage and tumor infiltrating lymphocytes as prognostic factors in stage III colorectal cancers. *Journal of Gastroenterology and Hepatology* **37** (2022) 551–557. doi:10.1111/jgh.15774.

69 .Zhao Z, Zhang X, Li Z, Gao Y, Guan X, Jiang Z, et al. engAutomated assessment of DNA ploidy, chromatin organization, and stroma fraction to predict prognosis and adjuvant therapy response in patients with stage II colorectal carcinoma. *American Journal of Cancer Research* **11** (2021) 6119–6132.

70 .Jones HJS, Cunningham C, Askautrud HA, Danielsen HE, Kerr DJ, Domingo E, et al. engStromal composition predicts recurrence of early rectal cancer after local excision. *Histopathology* **79** (2021) 947–956. doi:10.1111/his.14438.

71 .Miller S, Bauer S, Schrempf M, Schenkirsch G, Probst A, Märkl B, et al. engSemiautomatic analysis of tumor proportion in colon cancer: Lessons from a validation study. *Pathology, Research and Practice* **227** (2021) 153634. doi:10.1016/j.prp.2021.153634.

72 .Li T, Yu Z, Yang Y, Fu Z, Chen Z, Li Q, et al. engRapid multi-dynamic algorithm for gray image analysis of the stroma percentage on colorectal cancer. *Journal of Cancer* **12** (2021) 4561–4573. doi:10.7150/jca.58887.

73 .Wright AI, Dunn CM, Hale M, Hutchins GGA, Treanor DE. engThe Effect of Quality Control on Accuracy of Digital Pathology Image Analysis. *IEEE journal of biomedical and health informatics* **25** (2021) 307–314. doi:10.1109/JBHI.2020.3046094.

74 .Zhao K, Li Z, Yao S, Wang Y, Wu X, Xu Z, et al. engArtificial intelligence quantified tumour-stroma ratio is an independent predictor for overall survival in resectable colorectal cancer. *EBioMedicine* **61** (2020) 103054. doi:10.1016/j.ebiom.2020.103054.

75 .Martin B, Banner BM, Schäfer EM, Mayr P, Anthuber M, Schenkirsch G, et al. engTumor proportion in colon cancer: results from a semiautomatic image analysis approach. *Virchows Archiv: An International Journal of Pathology* **477** (2020) 185–193. doi:10.1007/s00428-020-02764-1.

76 .Geessink OGF, Baidoshvili A, Klaase JM, Ehteshami Bejnordi B, Litjens GJS, van Pelt GW, et al. engComputer aided quantification of intratumoral stroma yields an independent prognosticator in rectal cancer. *Cellular Oncology (Dordrecht, Netherlands)* **42** (2019) 331–341. doi:10.1007/s13402-019-00429-z.
